# How deceptive are microstructures in granitic rocks? Answers from integrated physical theory, phase equilibrium, and direct observations

**DOI:** 10.1007/s00410-018-1488-8

**Published:** 2018-07-20

**Authors:** M. B. Holness, J. D. Clemens, R. H. Vernon

**Affiliations:** 1 Department of Earth Sciences, University of Cambridge, Downing St, Cambridge, CB2 3EQ UK; 2 Department of Earth Sciences, University of Stellenbosch, Private Bag X1, Matieland, 7602 South Africa; 3 Department of Earth and Planetary Sciences, Macquarie University, Sydney, NSW 2109 Australia

**Keywords:** Granitic rocks, Textural development, Microstructure, Textural equilibration, Recrystallisation, Textural modification

## Abstract

In this contribution, we address the vexed question of the extent to which microstructures in granitic rocks reflect their igneous histories or have been masked by later events. The previous works have tended to address the problem either using theoretical or modelling considerations, or by interpretation of observed microstructures. Here, we use an approach that integrates the theory of microstructural development and the results of experimental phase-equilibrium studies with direct observation of natural examples on a variety of scales. We show that the predictions of the theoretical and experimental approaches agree perfectly with the mesoscopic and microscopic evidence from granitic rocks themselves. Our conclusion is that although, in many cases, granitic rock microstructures have been modified by near-solidus reactions and crystallisation, in the absence of tectonic deformation the fundamental elements of their igneous heritage remain intact. This means that it is perfectly in order to infer aspects of crystallisation sequences, magmatic reactions, and magma flow through careful microstructural observations. Thus, our answer to the question of how deceptive granitic textures are is, in most instances, ‘not very’. However, some undeformed plutons have undergone fluid-driven alteration, and others have been affected by contact metamorphism. Thus, each case should be examined on its own merits.

## Introduction

Some petrologists hold that the protracted cooling histories of plutonic magmas, both above and below their solidi, mean that the primary textures formed during their solidification have been sufficiently modified that they are now ‘untrustworthy’ and cannot be used to interpret igneous crystallisation histories (e.g., McBirney 2009; Glazner and Boudreau 2011; Higgins 2011; Glazner et al. 2017; Bartley et al. 2018). Accordingly, it is assumed that subsolidus adjustments, such as recrystallisation, deformation, and the growth of secondary minerals, have produced what amount to metamorphic textures (c.f. Means and Park 1994). For example, Coleman et al. (2005, p. 1) stated that “thermal models indicate that slow incremental growth can maintain much of a pluton at temperatures corresponding to the amphibolite facies of metamorphism for extended time periods” promoting “subsolidus textural modification that is likely to further obscure any primary intrusive contacts between intrusive increments.” Similarly, McBirney (2009, p. 4) wrote that “the textures of most coarse-grained igneous rocks are essentially metamorphic … As they cool, the minerals pass through a range of conditions in which a metamorphic petrologist would normally expect there to be conspicuous changes, including both compositional and textural re-equilibration of the kind seen in other rocks under comparable conditions.” A close reading of this statement reveals that its author is suggesting that the plutonic systems have remained at high temperatures for periods comparable to those that result in substantial modification of microstructures formed during metamorphic reactions. The loss of the microstructural record of mineral growth and dissolution in rocks as coarse-grained as most granites is encountered only in high-grade mid-to-deep-crustal rocks such as granulites and some amphibolites. Do plutons really linger at high temperatures for comparable times? Are the microstructures in plutonic igneous rocks comparable to those in granulites? A simple calculation casts doubt on this suggestion. Granitic magmas emplaced into the shallow crust typically have liquidus temperatures around 900 °C. Granitic sheets 1 km thick would cool to their solidi on time scales of the order of 10^3^ years and reach ambient wall-rock temperatures in times of the order of 10^4^ years (e.g. Bea 2010). Such time scales are orders of magnitude shorter than those of regional tectonism and metamorphism, which are typically of the order of 10^6^–10^7^ years (e.g., England and Thompson 1984).

In contrast, Vernon and Paterson (2008) and Vernon (2010) have argued that, although minor grain-boundary adjustments are common during the last stages of crystal growth (together with intragranular changes such as exsolution and deuteric alteration), extensive grain-shape changes such as those suggested by McBirney (2009) and Coleman et al. (2005) typically do not occur in granitic rocks unless they have been subjected to tectonic deformation or superimposed metamorphism. Thus, unlike rapidly cooled lavas and pyroclastic rocks, ‘metamorphic’ effects are present in medium-to-coarse-grained granitic rocks, and the debate is essentially over the extent to which these effects obliterate the record of the original igneous crystallisation.

Given the several recent studies that advocate a significant overprinting of primary igneous microstructures in plutonic igneous rocks, it is appropriate to re-examine what is currently known about microstructural evolution, to assess how likely it is that sustained, slow magmatic, and subsolidus cooling could obscure igneous crystallisation relations. Here, we summarise previously presented arguments, and develop further lines of evidence to support our interpretation that plutons preserve sufficient microstructural information to permit, through comparison with experimental and modelling determinations of crystallisation phase relations, the inference of pressures and temperatures of crystallisation, and volatile contents of the magmas and parameters such as oxygen fugacity. As a first step, we present a review of the theoretical framework, together with some illustrations of the predicted phenomena. Following that, we examine the evidence preserved in rocks, in the context of the established theoretical framework. Finally, we examine the extent to which the predictions of phase-equilibrium studies mirror the textures that we can actually observe in granitic rocks.

## Microstructures in solidifying systems without chemical reactions or deformation

Microstructures in non-deforming igneous rocks that have not undergone any chemical reactions to form secondary minerals fall on a spectrum between two end-member states. The first is that formed entirely by crystal growth and reaction, resulting in what would generally be described as a primary igneous microstructure. The second is that in which the microstructure, in either the hyper- or subsolidus, is governed entirely by the minimisation of internal energies, commonly described as being in textural equilibrium (although, in practice, true and complete equilibrium cannot be attained; it is only approached to various degrees). Textural equilibration results in the modification, by diffusive mass transport during static recrystallisation, of microstructures formed by chemical reaction (i.e., crystal growth). Subsolidus textural equilibration of microstructures dominated by the effects of chemical reaction therefore requires long periods spent at high temperature and creates what is essentially a metamorphic microstructure. The problem outlined in the introduction can therefore be reduced to the question of precisely where the microstructures in fully solidified igneous rocks lie within this spectrum of states.

The underlying control on microstructural evolution, and the position of any rock on the spectrum, is essentially the extent to which the system departs from chemical equilibrium. For large degrees of undercooling (defined as the difference between the equilibrium liquidus temperature and ambient temperature in a solidifying system), microstructures result entirely from reaction (i.e., crystal growth). In this regime, grain size is determined by the balance between nucleation and growth, while grain shape is determined by the extent to which growth is diffusion-limited (for large degrees of undercooling) or interface-controlled (for smaller degrees of undercooling) (e.g., Lofgren 1974; Fenn 1977; Donaldson 1976; Faure et al. 2003). Conversely, since the driving force for textural equilibration is small, it is only when the departure from chemical equilibrium is also very small, or non-existent, that microstructures formed during reaction can evolve toward textural equilibrium, at either hypersolidus or subsolidus conditions.

Textural equilibration occurs by diffusion, so grain size and thermal history are critical parameters that govern microstructural evolution toward textural equilibrium. Hence, the rocks that are most likely to have microstructures controlled mainly by the kinetics of crystal growth during solidification (i.e., chemical reaction) are those that crystallised rapidly, with fast subsolidus cooling, or those that solidified rapidly enough to grow euhedral grains but sufficiently slowly that the resultant rock is coarse-grained. Conversely, rocks that are most likely to have microstructures controlled mainly by the minimisation of interfacial energies are either fine-grained rocks that remained at high temperatures for a significant period (permitting the attainment of subsolidus textural equilibrium), or rocks that solidified at sufficiently low degrees of undercooling that textural equilibration could keep pace with crystal growth (permitting the microstructure to remain in textural equilibrium as it cools through the hyper- and subsolidus). Since granitic plutons are both coarse-grained and relatively slowly cooled, they are likely to display some characteristics of both end-member states.

### Microstructures controlled by crystal growth

During solidification at rates typical of plutonic magmas (as mentioned above), the initial degree of undercooling is generally sufficiently small that grain growth is controlled by the kinetics of attachment at the solid–liquid interface. Under such conditions, the shapes of grains are determined primarily by the relative rates of growth of the different faces and the details of the attachment kinetics. Some minerals, such as plagioclase, generally grow with atomically smooth interfaces by a birth-and-spread mechanism (Ohara and Reid 1973), while others, such as quartz, grow with atomically rough interfaces, in at least some crystallographic directions, resulting in grains with some areas of rounded surface at the optical scale (Kirkpatrick 1975, 1981). Recent work has demonstrated that the shapes of isolated plagioclase grains, growing from a melt under conditions of interface control, vary systematically with crystallisation rate (Holness 2014).

**Fig. 1:**
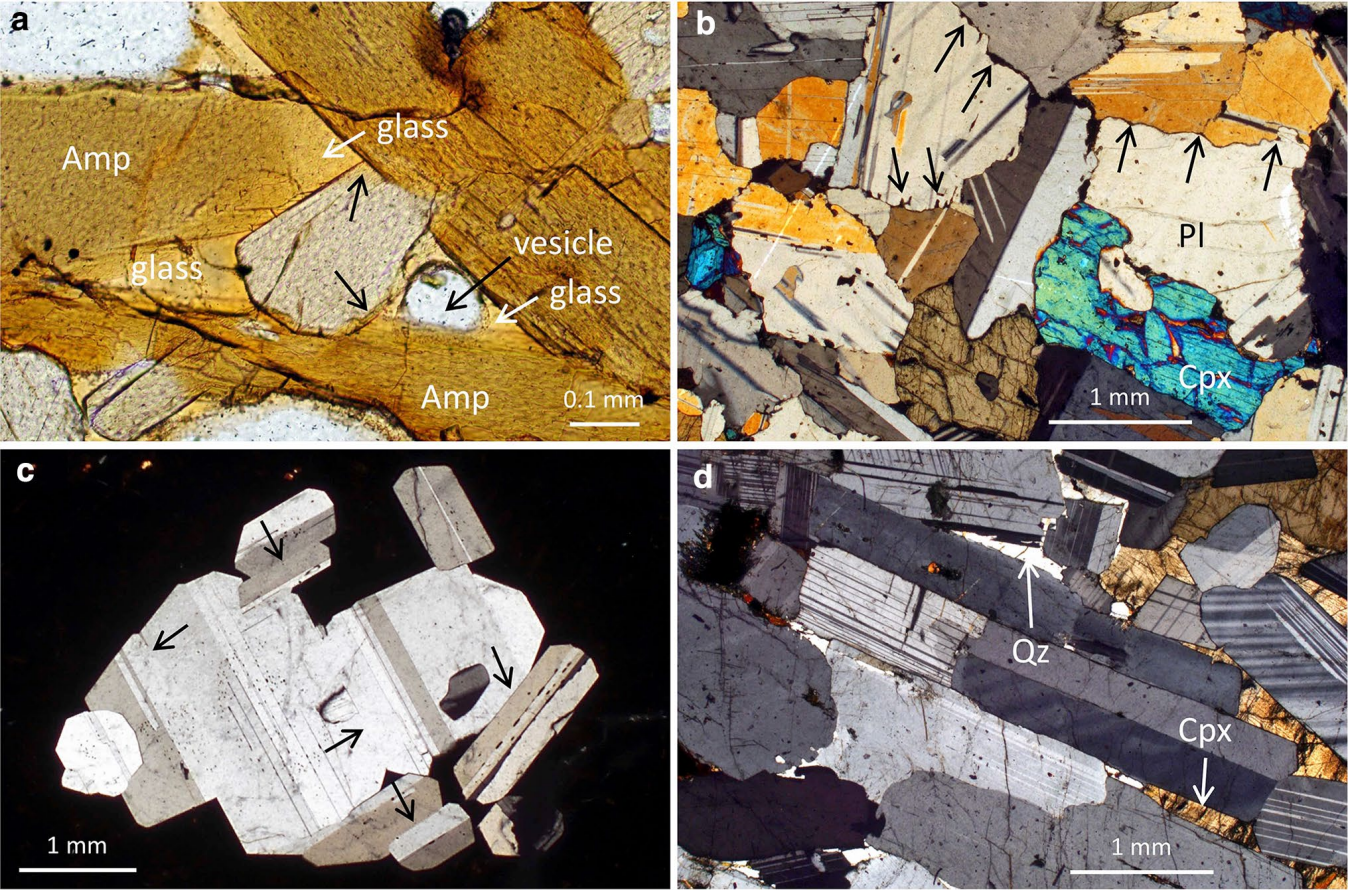
**a** Glassy nodule entrained in alkali basalt, Kula, Turkey (previously described by Holness et al. 2005). The dominant mineral is kaersutitic amphibole (Amp), set in vesicular glass (with examples of the glass and vesicles also indicated). Grain junctions are formed of the intersection of planar interfaces, with no evidence of modification towards the equilibrium melt–solid–solid dihedral angle (examples are arrowed). Plane-polarised light. **b** Gabbroic nodule entrained in the 1950 flow of the Kameni Islands, Santorini, Greece (previously described by Holness et al. 2007), comprising plagioclase (Pl) and augite (Cpx). Note the highly irregular grain boundaries separating the low aspect ratio (denoting slow cooling) plagioclase grains. The irregularities form pockets filled with glass (examples are arrowed), demonstrating that they are a consequence of primary solidification. Crossed polars. **c** Polycrystalline aggregate of plagioclase in a porphyritic basalt from the FAMOUS area of the Mid-Atlantic Ridge [sample AII-77-38-11 of Bryan (1979), and sample number 126478 of the Harker Collection, University of Cambridge]. The grain boundaries in this cluster are generally planar and parallel to commonly developed growth faces in plagioclase (examples are arrowed). Crossed polars. **d** Plagioclase-rich upper part of a modally graded trough layer from the Skaergaard Intrusion, East Greenland comprising strongly aligned euhedral plagioclase grains with interstitial quartz (Qz) and augite (Cpx), indicated by arrows. These layers are argued to be analogues of sedimentary deposition features (Irvine 1983; Irvine and Stoeser 1978; Vukmanovic et al. 2018), but see Glazner (2014). Note the planar grain boundaries formed by the juxtaposition of (010) faces of adjacent plagioclase grains. Crossed polars. Sample 48969 from the Harker Collection, University of Cambridge

As solidification progresses, and grains become larger and more numerous, they eventually impinge. When this occurs, the angle formed by the meeting of the two interfaces is a result of the random relative orientations of the two impinging grains (Holness et al. 2005) (Fig. 1a).

Further growth results in the formation of a grain boundary between the two impinging grains, and the morphology of these boundaries is characteristic (Fig. 1b); they are typically oriented at high angles to commonly developed growth faces, may be highly irregular, and may also contain small inclusions of other minerals (Holness et al. 2007). Conversely, boundaries formed by synneusis (during which previously isolated grains are brought together) are parallel to the growth faces of the two grains (e.g., Vance 1969; Schwindinger and Anderson 1989; Schwindinger 1999; Graeter et al. 2015), and are, therefore, generally planar (Fig. 1c). Planar grain boundaries, parallel to growth faces in the grains on either side (Fig. 1d), are also common in rocks in which mineral grains were re-arranged during magmatic flow (e.g., Vukmanovic et al. 2018). Thus, in a fully solidified rock, the relative orientations and morphologies of grain boundaries depend on the relative importance of mechanical re-arrangement, synneusis, and static growth to impingement during solidification.

The grain-size distributions created during primary solidification depend on a multitude of processes (e.g., Marsh 1988, 1998). In a closed system, the grain-size distribution depends on the balance between growth and nucleation, whereas, in an open system, specific grain-size fractions can be lost or gained. The process of Ostwald ripening involves the dissolution of small grains, with their components feeding growth of larger grains, driven by the difference in stability created by the differences in the curvature of their solid–liquid interfaces. Critically, in solidifying systems, Ostwald ripening is generally insignificant, because it is driven only by a reduction in the total interfacial energy, and this driving force is generally much smaller than the thermodynamic drive for crystallisation. Since the surface area is increasingly important as grains become smaller, Ostwald ripening when it does occur is significant only for very small grains (Cabane et al. 2001, 2005).

### Microstructures controlled by the minimisation of interfacial energies

**Fig. 2:**
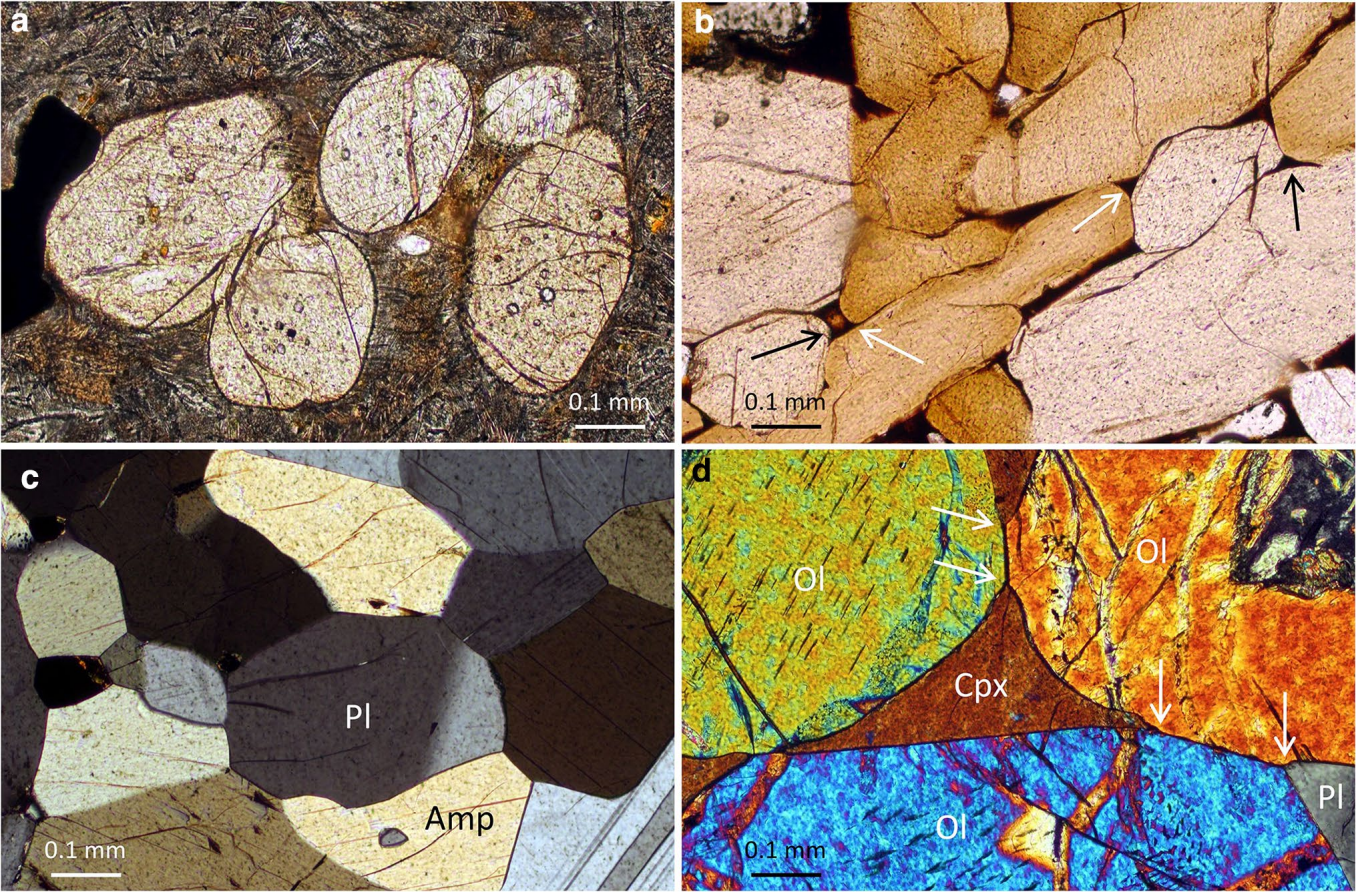
**a** Cluster of pyroxene phenocrysts in a basic sill, Isle of Mull, Scotland. Note the unusual rounded shapes of the grains, indicating a significant approach to a minimum-energy shape, facilitated by a long period at low-to-zero undercooling. Plane-polarised light. Sample 13483 from the Harker Collection, University of Cambridge, previously described by Anderson and Radley (1915). **b** Glassy nodule entrained in an alkali basalt from Kula, Turkey (previously described by Holness et al. 2005). The dominant mineral is kaersutitic amphibole, set in (dark) vesicular glass. Melt–solid–solid junctions are formed by the meeting of smoothly curved melt–solid interfaces, forming the equilibrium melt–solid–solid dihedral angle (examples arrowed). Note that the smallest amphibole grains are more rounded than the larger grains, which preserve large areas of planar growth faces, indicating a limited approach to the minimum-energy shape. Plane-polarised light. **c** Nearly bimineralic amphibolite with plagioclase (Pl) and amphibole (Amp) grains. The grain size is unimodal, grains have smoothly curved boundaries, and dihedral angles are high, all indicating a close approach to subsolidus textural equilibrium. Crossed polars. **d** Olivine (Ol) cumulate from the Isle of Rum (Scottish Inner Hebrides) with interstitial augite (Cpx) and plagioclase (Pl). Note the abrupt change in curvature of the olivine-augite and olivine-plagioclase grain boundaries in the vicinity of three-grain junctions (arrowed). This is caused by a limited approach to subsolidus textural equilibrium, as the dihedral angle increases from the original geometry inherited by the augite and plagioclase that pseudomorphed the pre-existing, melt–olivine–olivine dihedral angle. Crossed polars

Since the curvature of interfaces (grain boundaries, fluid–solid, or melt–solid interfaces) is associated with a chemical potential (Thomson 1871; Bulau et al. 1979), textural equilibrium (at least for isotropic materials) requires that all interfaces have a constant mean curvature. For isolated crystals with complete isotropy of surface energy, this means that the lowest energy shape is a sphere, although the anisotropy of interfacial energies for all substances of geological interest means that the minimum-energy shape of mineral grains isolated in a liquid involves some planar areas of interface joined by smoothly curved areas (Herring 1951, e.g., Fig. 2a).

For polycrystalline materials, the demand of interfacial energy minimisation required for textural equilibrium means that interfacial energies must balance at all intersections. This leads to attainment of the equilibrium dihedral angle at all three-grain junctions and at all pore corners (Fig. 2b, c). In such a system, the geometry of melt-filled pores is controlled by the porosity and the equilibrium melt–solid–solid dihedral angle, with complete connectivity attained for angles < 60° in fully isotropic systems. As reviewed by Holness (2006), median melt–solid–solid dihedral angles are commonly < 60° in silicate systems, but the anisotropy of common rock-forming silicate minerals means that melt connectivity may not be complete at low porosities (Minarik and Watson 1995; Laporte and Provost 2000).

For rock-forming minerals, solid–solid–solid dihedral angles are close to 120° (reviewed by Holness 2010). This means that, in (or close to) subsolidus textural equilibrium, large grains are necessarily bounded by interfaces that are concave outward, while smaller grains are bounded by interfaces that are concave inward. In texturally equilibrated, subsolidus, monomineralic crystalline aggregates, grain boundaries migrate toward their centres of curvature, with the result that large grains grow at the expense of small grains, and the system attains a constant normalised grain-size distribution. The average grain size will continue to increase if the temperature remains sufficiently high to permit grain-boundary migration. In the material science literature, this process is generally known as ‘normal grain growth’ and only occurs in materials in which the grain-boundary curvature is determined by interfacial energy minimisation; it typically occurs in quartz–feldspar aggregates in high-grade metamorphic rocks.

## How does textural equilibration modify igneous microstructures?

Microstructures created during solidification are generally controlled by kinetic factors, such as the kinetics of attachment and diffusion. This is because the thermodynamic driving forces for solidification are generally much greater than those driving the minimisation of interfacial energy. Textural equilibration and the reduction of interfacial energy can only begin once the undercooling has been reduced, so that grain growth is slowed or stopped, for example during a cooling hiatus (as shown by the isothermal experiments of Means and Park 1994) or when the rock is fully solidified.

In partially crystallised rocks, microstructural modification driven by interfacial energy minimisation (i.e., textural equilibration) begins at melt-filled pore corners that are formed at junctions between two grains. In contradiction to the commonly used model of Mullins (1957), detailed observations of glassy enclaves in mafic volcanic rocks (Holness et al. 2005) have shown that the equilibrium solid–solid–melt dihedral angle at pore corners is established by the progressive rotation of large areas of grain boundary in the vicinity of the junction. Thus, the median dihedral angle varies smoothly during textural equilibration, from its original (and highly variable) value created during grain impingement, toward the commonly lower (and less variable) values typical of solid–melt equilibrium. Rotation of the grain boundaries in the vicinity of the pore corner alters the curvature of the solid–melt interfaces further from the junctions, and this change in curvature propagates outward until a constant mean curvature is attained (Holness and Siklos 2000). These microstructural changes occur by diffusion within the melt.

As solidification proceeds in a crystal mush, the melt-filled pores can be filled by grains of a different mineral, forming two-phase dihedral angles. The final geometry of the resultant three-grain junctions depends on the relative growth rates of the minerals involved (Holness 2015). For the case in which the interstitial mineral grows faster than the framework-forming mineral, the interstitial mineral pseudomorphs the shape of the melt-filled pore, inheriting the melt–solid–solid dihedral angles (e.g., Holness and Clemens 1999; Sawyer 2001; Rosenberg and Riller 2000; Marchildon and Brown 2002; Holness and Sawyer 2008; Holness 2015). At textural equilibrium, the dihedral angles formed at the junctions of three solid grains are ~ 120°, significantly higher than most of those generated in a melt-bearing aggregate by impingement or as subsequently modified by hypersolidus textural equilibration. In the subsolidus regime, textural equilibration, therefore, generally involves an increase in dihedral angles at three-grain junctions, and this also occurs by progressive rotation of large areas of interface. In this case, however, the interfaces involved are all grain boundaries (Fig. 2d). In monomineralic systems, the establishment of equilibrium interfacial curvature and equilibrium dihedral angles simply involves the migration of mass across grain boundaries, and so the time required to equilibrate monomineralic regions of any rock is significantly shorter than that required to equilibrate the polymineralic regions, in which equilibration requires the migration of mass along as well as across grain boundaries. Thus, at any time instant during equilibration in a polymineralic rock, the progress of subsolidus textural equilibration not only proceeds at a much slower pace than hypersolidus textural equilibration, but also varies locally, on a small scale.

The last element of the primary igneous microstructure to be lost during textural equilibration is the grain shape, as can occur during the high-grade metamorphism of felsic igneous rocks. Elongate or tabular shapes created during primary growth from the melt are generally retained after the equilibrium dihedral angle is fully established (Fig. 2b; Holness et al. 2005) and after constant mean curvature of grain boundaries has begun to be established. Since textural equilibration is a diffusive process, the larger grains take longer to attain the minimum-energy shape (Fig. 2b).

During textural equilibration, changes to the original grain-size distribution occur by several mechanisms. In fully solidified rocks, the process of normal grain growth occurs only after the establishment of the equilibrium dihedral angle, together with the minimum-energy curvature of the grain boundaries. In igneous systems that contain abundant melt, a hiatus in cooling can permit the onset of Ostwald ripening although, on geological timescales, this is only likely to be important for grains < 10 µm in size (Cabane et al. 2001, 2005). The agency of Ostwald ripening can be detected by considering grain shape as a function of size; dissolving grains tend to be rounded, whereas growing grains tend to be facetted, so Ostwald ripening results in a rounded shape for grains smaller than the critical radius (Holness 2018).

For a system in which crystals are in mutual contact, a loss of small grains can also occur by coalescence if the grain boundaries can sweep through the smaller grains (e.g., Schiavi et al. 2009, 2010), a process known as grain-boundary migration recrystallisation or ‘fast’ grain-boundary migration in deformed rocks metamorphosed at high temperatures and/or high H_2_O contents; see Vernon (2004) for a review. Fast grain-boundary migration has been suggested as an important syn-magmatic process in plagioclase-rich rocks undergoing mild deformation, since it requires only very small gradients in plastic strain energy (LaFrance et al. 1996). However, for geological materials, the rates and wider prevalence of this process are poorly understood.

## How does deformation modify microstructures?

Deformation is commonly invoked as an important process that drives recrystallisation and overprinting of original magmatic microstructures. Gravitationally-driven compaction is the mechanism most commonly cited as a driver for recrystallisation in mafic rocks (e.g., Meurer and Boudreau 1998; Boorman et al. 2004) although the arguments set out by Holness et al. (2017) suggest that gravitationally-driven compaction is highly unlikely to be important in granitic magmas, in which any crystal mush is dominated by low-density minerals such as quartz and feldspar. Compaction in granitic rocks may occur by lateral magmatic flow, which can result in shear-enhanced compaction at channel margins (Rutter and Neumann 1995; Rabinowicz and Vigneresse 2004) or by filter pressing under the influence of fluid (gas) pressure (Sisson and Bacon 1999).

Deformation occurs by a range of processes, including dislocation creep, dissolution–reprecipitation, and melt-enhanced grain-boundary sliding (e.g., Paterson 1995, 2001), and the deformation mechanism depends on grain size, strain rate, and the amount of melt present. Significant changes in grain shape and size, or the creation of preferred orientations, require either dissolution–reprecipitation or the dynamic recrystallisation that accompanies dislocation creep.

The increase in internal energies associated with the creation of abundant lattice defects during dislocation creep drives recrystallisation, leading to the migration of grain boundaries and the formation of defect-free neoblasts. If the temperature remains sufficiently high, these neoblasts will grow and replace the original highly deformed grains. The end result is a rock with a strong preferred crystallographic orientation that corresponds to the dominant dislocation slip system. This well-understood process results in easily recognisable microstructures that are commonly observed in oceanic gabbros (e.g., Satsukawa et al. 2013).

Some authors advocate the overprinting of original microstructures during deformation by the preferential dissolution of unfavourably oriented grains and their subsequent recrystallisation in more favourable orientations (e.g., Boorman et al. 2004). This is essentially pressure-solution although, in the absence of dislocation creep, the development of a crystallographic preferred orientation by such a process necessitates anisotropy of dissolution and reprecipitation. This has been documented for plagioclase in metamorphic rocks, and results in fabrics defined by plagioclase grains that are elongate perpendicular to the (010) twins (Heidelbach et al. 2000; Arvidson et al. 2004; Svahnberg and Piazolo 2010). Such fabrics are not generally observed in plutonic rocks, in which plagioclase crystals are instead generally elongated parallel to (010).

## What microstructures are observed in granitic rocks?

The preceding sections lay out what is known about microstructural development in solidifying polycrystalline materials. The processes described, and the physics controlling them, are well understood and are not in question. We now turn attention toward the microstructures that are actually preserved in granitic rocks, and the extent to which they are controlled by crystal growth or by textural equilibration both above and below the solidus.

### How are granitic plutons constructed?

The physical and temporal intrusion history of a granitic pluton is critical in determining its thermal history and, therefore, the extent to which primary igneous microstructures might be overprinted in the subsolidus regime. Granitic plutons are commonly considered to be constructed by multiple, pulsed additions of magma over protracted geological periods (e.g., Coleman et al. 2004; Clemens et al. 2017a, b), with the lengthy cooling history of a large incrementally grown mass of magma providing the basis of arguments that plutonic microstructures must have undergone a significant subsolidus textural modification.

A different idea, which has gained substantial currency, is that some granitic plutons represent the unerupted cumulate mushes that remained after the withdrawal of more silicic, eruptible liquids to form rhyolitic volcanic rocks (e.g., Bachmann and Bergantz 2004, 2008; Deering et al. 2011; Glazner et al. 2015; Keller et al. 2015). Keller et al. (2015) assumed that all felsic magmas are produced by fractionation of mafic parents. However, it is demonstrable that many (most?) silicic magmas are the products of crustal melting, or have dominant crustal components (e.g., Nédélec and Bouchez 2015 and references therein), so comparisons of supposed fractionation trends are flawed. As summarised in Clemens and Stevens (2016), the idea that volcanic calderas were always underlain by residual plutons was actually questioned by Lundstrom and Glazner (2016), with the ‘evidence’ found to be more hypothesised than actual (e.g., Bagdonas et al. 2016). If this second, now apparently ruling, paradigm were correct, felsic plutonic rocks should be somewhat more mafic than their supposed complementary volcanic fractions, but this is not supported by the statistical evidence. The average Phanerozoic felsic volcanic rock (from the population with > 69 wt% SiO_2_) has markedly higher TiO_2_, MgO, FeO, and Mg#, and much lower K_2_O than the average Phanerozoic felsic plutonic rock (Condie 1993). Streck (2014) also criticised the mush paradigm using a battery of chemical arguments to address the infeasibility of plutons having spawned neighbouring volcanic accumulations. Furthermore, in many cases, it appears that granitic plutons are constructed from numerous sheet-like injections of magma, and the hiatuses separating individual injections could permit significant cooling and crystallisation (e.g., Horsman et al. 2009; Miller et al. 2011). Indeed, in some cases, the emplacement ages of the plutonic and volcanic chains in continental arcs are tens of millions of years apart, e.g., in the Peruvian Andes (Petford and Atherton 1995). These facts suggest that, in general, granitic plutons should be viewed neither as huge pools of slowly crystallising liquid magma, nor as the mushy residues left behind after removal of fractionated liquids. The corollary of this is that the notion of comprehensive microstructural re-equilibration should be examined critically and on a case-by-case basis, in the light of evidence from the rocks themselves.

### Where do granitic rocks lie on the spectrum between growth and equilibrium microstructures?

**Fig. 3:**
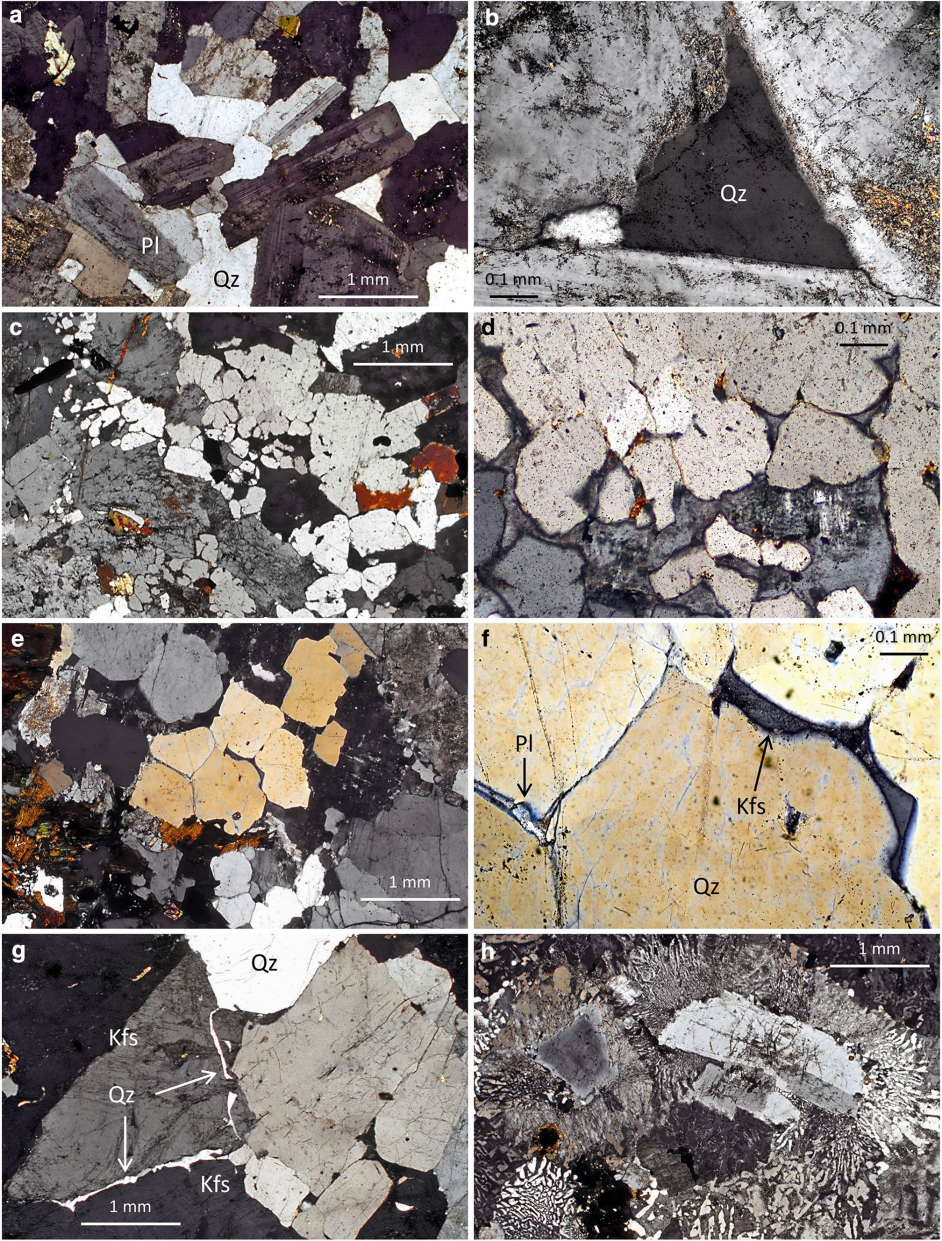
All images crossed polarised light. **a** Quartz–biotite–hornblende monzonite from Ben Nevis, Scotland (sample 14105 from the Harker Collection, University of Cambridge), showing well-preserved euhedral plagioclase grains with random orientation, with interstitial quartz. **b** Higher magnification view of **a** showing the shape of an interstitial quartz grain. The geometry of the pore corners is dominated by the impingement of the plagioclase grains, with slight modifications that are probably due to simultaneous growth of feldspar and quartz during the late stages of solidification. **c** Granite from Marsco, Skye (Scottish Inner Hebrides), showing a fine network of cuspate feldspar grains interstitial to rounded grains of quartz. Such features indicate relatively early crystallisation of quartz, followed by nucleation and growth of feldspar in a system that was approaching melt–solid textural equilibrium. Sample 124523 from the Harker Collection, University of Cambridge. **d** Close-up of an area of c, showing the complex intergrowth of feldspar and quartz. Note the low dihedral angles and the cuspate shape of the minor feldspar component, which suggests pseudomorphing of originally texturally equilibrated melt-filled pores between the rounded grains of quartz. **e** Granite from Virolahti, South Finland. Sample 87817 from the Harker Collection, University of Cambridge. **f** Higher magnification view of e, showing cuspate interstitial feldspar grains (Pl and Kfs, arrowed) that preserve a low feldspar–quartz–quartz dihedral angle. This is consistent with the feldspar having pseudomorphed a previously melt-filled pore space, close to hypersolidus textural equilibrium. **g** Granite from Coire Lan, Arran, Scotland, showing an irregular film of quartz (Qz, arrowed) separating alkali-feldspar grains (Kfs). Such a feature suggests that quartz infilled a grain-boundary melt film with irregular margins. **h** Hornblende granite, Skye (Scottish Inner Hebrides), dominated by granophyric intergrowths that nucleated on and grew away from earlier crystallised euhedral grains of plagioclase

The arguments of Coleman et al. (2005), that granitic microstructures are modified during long periods at high temperatures, can be tested by determining whether granitic rocks record a microstructural evolution from features created during crystal growth toward those that indicate textural equilibration. During recrystallisation driven by the minimisation of interfacial energies, the ‘canary in the coal mine’ is the dihedral angle, as this is the first part of the microstructure that will change. If the geometry of three-grain junctions that involve two phases is either close to that expected for grain impingement (i.e., formed by the meeting, at a wide range of angles, of planar grain boundaries defined by growth faces; Figs. 1a, 3a, b) or similar to that expected for the pseudomorphing of pore geometries controlled by melt–solid–solid equilibrium (i.e., formed by the meeting of smoothly curved grain boundaries, creating a narrow range of angles with a low median; Figs. 2a, b, d, 3c, d; Holness 2006; Holness and Sawyer 2008), there can have been only trivial amounts of textural equilibration at subsolidus conditions. A preliminary examination of a broad range of granitic rocks reveals that, while monomineralic quartz regions do commonly exhibit dihedral angles of ~ 120°, those at three-grain junctions that involve two minerals are generally far from 120° (Fig. 3a–d), consistent with insignificant extents of subsolidus textural equilibration. Thin films of a second phase commonly occur along grain boundaries (Fig. 3e–g). By analogy with similar features in migmatites that have been interpreted as pseudomorphs of melt (Platten 1982, 1983; Riller et al. 1996; Holness and Clemens 1999; Sawyer 2001; Rosenberg and Riller 2000; Marchildon and Brown 2002; Holness and Sawyer 2008), we suggest these formed by the late-stage infilling of grain-boundary melt films. They are typified by low dihedral angles, despite their high surface areas, which should impart a large driving force for textural equilibration (Fig. 3d, f, g). The presence of low dihedral angles associated with cuspate interstitial grains (Fig. 3d, f) demonstrates that some textural equilibration occurred in the hypersolidus, and we would expect this to be particularly important in large, rapidly emplaced intrusions that crystallised slowly.

The retention of primary igneous geometries at three-grain junctions is consistent with the common presence of planar growth faces and euhedral shapes of early crystallising phases (e.g., Fig. 3a), since the approach of grain shapes toward those typical of textural equilibrium requires considerably more mass transport than does the establishment of equilibrium subsolidus dihedral angles. In granitic rocks, there is a little sign of the kind of granular microstructure that would indicate a close approach to subsolidus textural equilibrium (Vernon and Paterson 2008).

Fine-grained granophyric intergrowths are common in subvolcanic granitic intrusions (e.g., Fig. 3h) and also occur in some large batholiths. Their fine grain size creates a high interfacial area, which provides a significant driving force for the rounding of interfaces and grain growth. This process results in polygonal aggregates and rounded inclusions of quartz in feldspar, as observed in metamorphosed granophyric rocks (e.g., Vernon 2004, pp. 256–260, Fig. 4.65). Such features, indicative of recrystallisation, are not observed in granitic rocks, in which typical granophyric microstructures display well-developed angular morphologies that indicate crystal growth during eutectic-like co-crystallisation of quartz and feldspar.

The typical granitic microstructures illustrated in Fig. 3 are inconsistent with any modification of primary microstructures by interfacial energy minimisation. They would not be preserved in a rock that had undergone recrystallisation, and their persistence provides strong evidence that granite microstructures are indeed primary and have not been overprinted by any long sojourn at high temperatures. In addition to the above criteria for the recognition of extensive recrystallisation, the following also provide evidence that counter the idea of extensive grain-shape change in granitic rocks.

### Examples of the preservation of characteristic igneous microstructures in granitic rocks

**Fig. 4:**
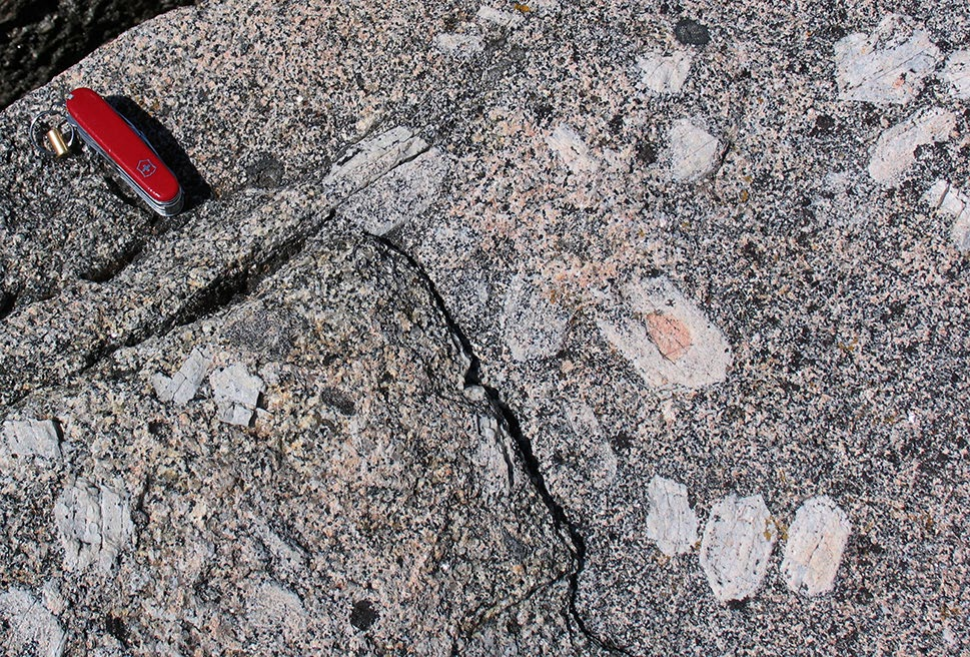
Euhedral megacrysts of K-feldspar, some zoned, some with crystallographically (zonally) arranged inclusion patterns, from the South Mountain Batholith of Nova Scotia, Canada. Knife 9 cm long

**Fig. 5:**
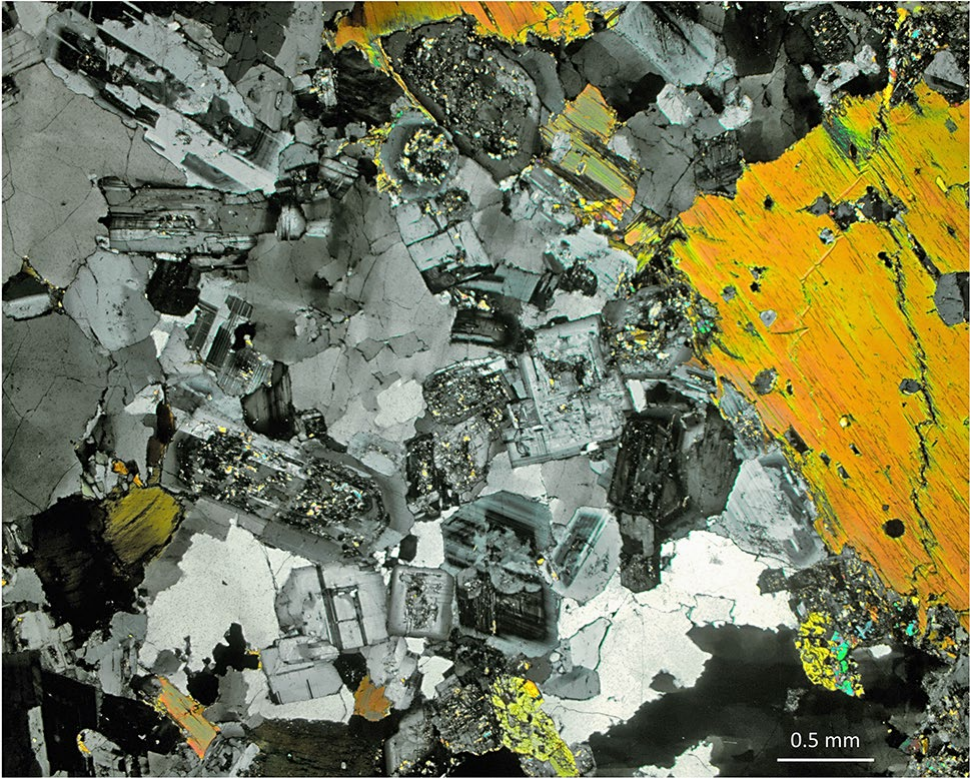
Predominantly euhedral crystals of plagioclase, many compositionally zoned, with biotite and interstitial quartz (some with subgrains resulting from weak solid-state deformation) in the Cootralantra Granodiorite from southeastern New South Wales, Australia. Crossed polars

**Fig. 6:**
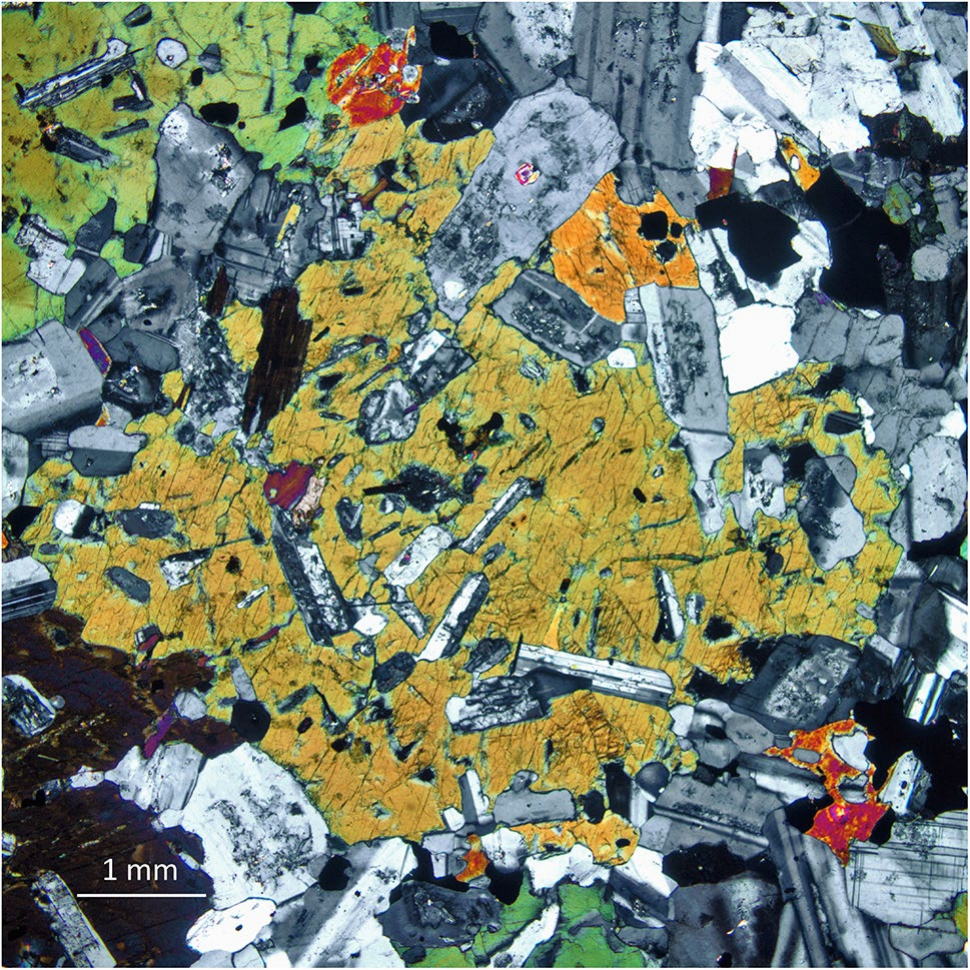
Predominantly euhedral inclusions of plagioclase crystals, many zoned, in hornblende in a diorite from the Halfmoon pluton on Stewart Island, New Zealand. Crossed polars

**Fig. 7:**
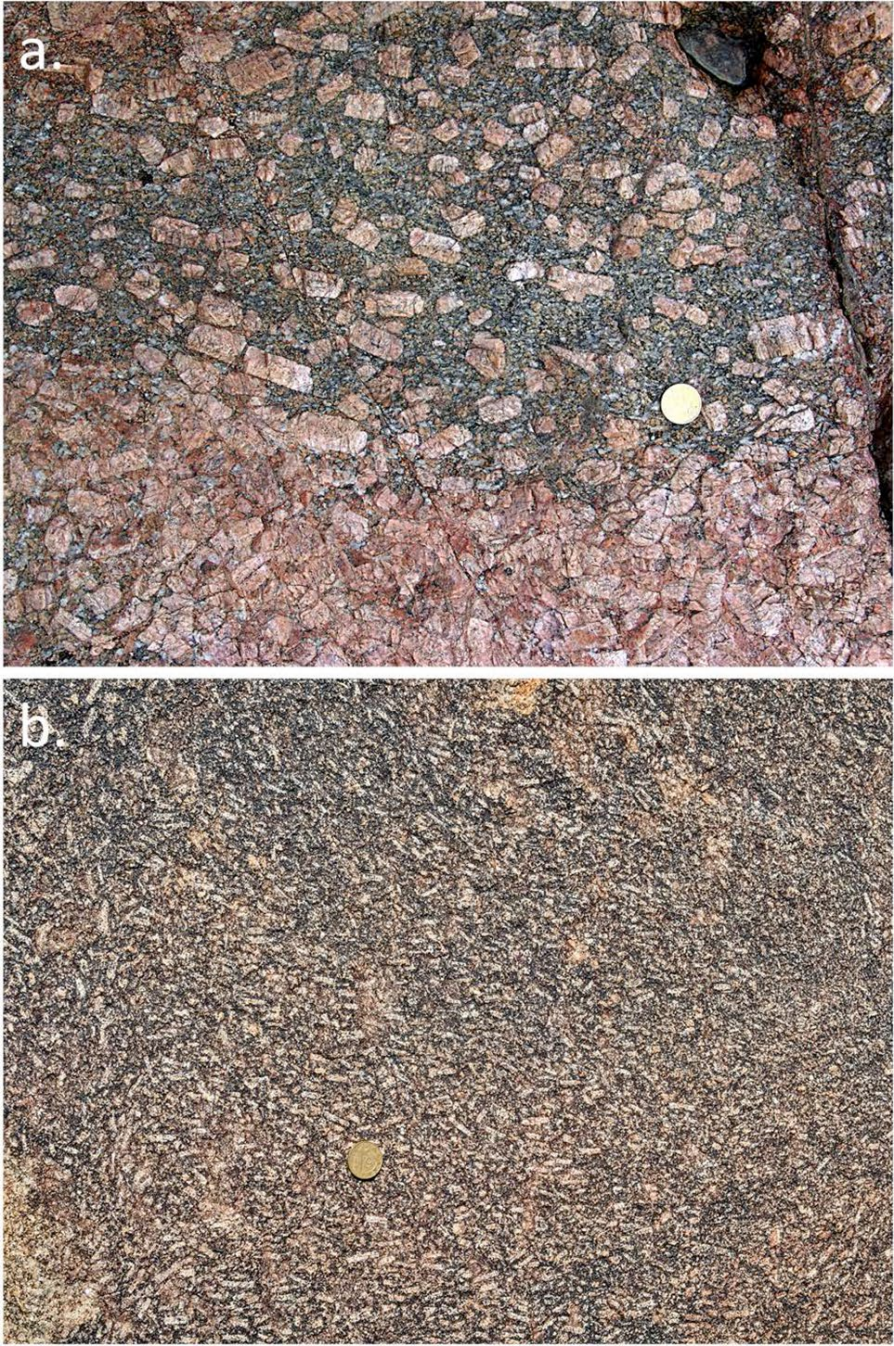
**a** Euhedral megacrysts of K-feldspar aligned in a magmatic flow foliation in a granitic rock, Corsica. The coin is 24.25 mm in diameter. **b** Chaotic magmatic flow patterns, defined by the orientations of relatively small K-feldspar phenocrysts in a monzogranite from the Donkerhuk batholith in Namibia (part of Fig. EA1.13 in Electronic Appendix 1 of Clemens et al. 2017b). The coin is 24.9 mm in diameter

Euhedral-to-subhedral feldspar or quartz grains (Figs. 4, 5, 6, 7), as well as anhedral, interstitial feldspar, and quartz (Figs. 3a–g, 5), are common in granitic rocks, but typically absent from metamorphosed granitic rocks, which are characterised by polygonal grains of these minerals (e.g., Vernon 1999). Similarly, plagioclase inclusions in quartz and K-feldspar, biotite, and hornblende (Fig. 6) are typically euhedral in granitic rocks, retaining shapes that indicate growth in a melt-rich environment. These contrast with the rounded shapes of inclusions of quartz in feldspar (and vice versa) in metamorphic rocks, which indicate an approach to textural equilibrium through the minimisation of the interfacial area of the inclusions. In addition, crystallographically (zonally) arranged inclusions are common in K-feldspar megacrysts in granitic rocks (Fig. 4), in contrast to randomly orientated and distributed inclusions in porphyroblasts from metamorphic rocks (Vernon 1999, 2004). The zonally arranged inclusions in the K-feldspar megacrysts are entirely consistent with a primary origin during solidification, and demonstrate that the megacrysts were not formed in the subsolidus regime, by metamorphic recrystallisation.

### Preservation of oscillatory zoning in crystals

During crystal growth in silicate liquids, oscillatory zoning can be caused by fluctuations in growth conditions during solidification or diffusion-controlled self-organisation (e.g., Haase et al. 1980; Ortoleva 1994; Perugini et al. 2005). Such zoning is particularly common in plagioclase, K-feldspar, and quartz, and some granitic rocks are characterised by complete oscillatory zoning patterns in all three of these minerals (e.g., Wiebe et al. 2007), as shown in Vernon (2010, Fig. 40). Indeed, as discussed by Vernon (2010), oscillatory zoning is also preserved in a wide range of other common minerals in granitic rocks, including zircon, allanite, titanite, apatite, cordierite, hornblende, and biotite, as well as muscovite (Roycroft 1991).

As we have seen above, recrystallisation generally involves grain-boundary migration, to minimise interfacial energies. Grain boundaries also migrate during normal grain growth. A further driver for grain-boundary migration is the reduction of lattice defects during the dynamic recrystallisation associated with dislocation creep. Most granitic rocks show a little evidence of significant subsolidus deformation, so the main drivers for grain-boundary migration are those associated with textural equilibration. Importantly, grain-boundary migration would truncate or obliterate oscillatory zoning, but this is not generally observed in granitic rocks (Vernon and Paterson 2008). Exceptions are provided by the uncommon instances of ‘contact melting’ that resulted from crystal impingement during growth above the solidus (e.g., Vernon et al. 2004, Fig. 30; Vernon 2010, Fig. 7) and internal pattern truncations that resulted from partial resorption in response to magma mingling/mixing (Vernon 2010, Fig. 6). The general preservation of zoning demonstrates that recrystallisation cannot have been pervasive.

### Phenocrysts, antecrysts, xenoliths, and xenocrysts

Many granitic rocks from batholithic bodies contain mesoscopic-to-microscopic crystals and crystal aggregates that differ texturally (and mineralogically) from the bulk of the enclosing rock. The most obvious of these features are the common, large, subhedral-to-euhedral alkali-feldspar crystals that some refer to as megacrysts, and which have been shown (Vernon 1986) to represent phenocrysts produced through igneous crystallisation. Figures 4, 7 show examples of these features, and demonstrate that these phenocrysts are aligned and, sometimes, concentrated by magmatic flow. Some of these concentrations of platy or tabular crystals show no evidence of deformation and yet contain crystals arranged in imbricate (or tiled) fashion. These alignments and imbrications result from magmatic flow, which can change direction on a decimetric-to-metric scale (e.g., Fig. 7b). This implies that the crystals existed independently of each other, in a sufficient volume of magmatic liquid to allow for passive and sometimes chaotic rotation (Paterson et al. 1989; Vernon 2000). Although there may be cases of K-feldspar coarsening through in situ growth, these phenocrysts cannot generally represent products of solid-state recrystallisation or growth driven by some kind of ‘thermal cycling’ (c.f. Johnson and Glazner 2010).

Many granitic rocks contain fine-grained xenoliths, some of which are quartzofeldspathic. Though not usually abundant, xenocrysts of quartz and feldspar, as well as many other minerals, are also common in granitic rocks, as are antecrysts (inherited from related magmas). Some of the xenoliths and xenocrysts are demonstrably derived from rocks that bear no relation to the immediate wall rocks intruded by the granitic rock that contains them. Indeed, many are of considerably deeper origin than the emplacement depth of the host granitic magma. As such, they have spent essentially the entire cooling history of the pluton encapsulated in hot magma and granitic rock. Some xenocrysts and antecrysts show reaction or dissolution textures, commonly with overgrowths of newly formed magmatic crystals. All these reaction textures are marked by fine grain sizes, disequilibrium microstructures (such as wormy or dactylitic resorption and fine embayments), together with low dihedral angles. Small, fine-grained, metamorphic xenoliths of deep origin (e.g., schistose metasedimentary rocks) retain their fine-grained microstructure, foliations, tiny porphyroblasts, etc. The constant factor linking all these features is the very low degrees of grain coarsening and textural equilibration. The above observations do not support the idea that the microstructures in the enclosing granitic rocks are greatly altered from their original igneous character, especially since fine-grained rocks should be the most susceptible to recrystallisation, coarsening, and textural equilibration.

### Preservation of microstructures in igneous enclaves

Fine-grained, intermediate-to-silicic rock enclaves (igneous, microgranular enclaves, microgranitoid enclaves, or ‘mafic enclaves’) in granitic rocks have been the subject of almost boundless petrogenetic interest. Irrespective of their origins, their textural features are highly instructive. As discussed and illustrated by Vernon (2014), the typical preservation of characteristic igneous microstructures in these enclaves provides strong evidence against extensive solid-state grain-shape modification in granitic rocks. As mentioned earlier, recrystallisation and grain growth would be expected to have had substantial effects on such fine-grained aggregates. In contrast to the recrystallised grain aggregates of enclaves that have unequivocally undergone metamorphism (i.e., a metamorphic event recorded both in the granitic rock and its surroundings, unrelated to any metamorphic event that might have been generated by the pluton itself), undeformed microgranitoid enclaves in granitic rocks typically preserve igneous features, such as euhedral phenocrysts, elongate plagioclase laths, interstitial or micropoikilitic quartz and/or K-feldspar, acicular apatite and zircon, and magmatic flow alignment of undeformed crystals, as well as mantled quartz and feldspar xenocrysts that reflect mechanical magma mixing (e.g., Vernon 2004, 2014).

## Phase petrology: predictions and physical proofs

Having surveyed a wide range of primary igneous microstructures preserved in granitic rocks, we now examine the evidence of that igneous character that comes from phase-equilibrium studies. Many aspects of textural development in magmatic systems are related to the order of appearance (crystallisation) and disappearance (through reaction or resorption) of minerals. The prediction of such relationships, through the construction of phase diagrams, is the province of experimental petrology. Since the 1950s, petrologists have noted the correspondence between what they have seen in the rocks (textures, reaction relationships, and apparent orders of crystallisation) and what has been predicted by experiments and, latterly, in calculated pseudosections. For granitic bulk compositions, such correspondence is remarkably good, and this suggests that the microstructures preserved in granitic rocks can be reliable indicators of crystallisation order and mineral reactions. The following examples illustrate the consonance of phase relations and textures in granitic rocks.

### Early formed plagioclase

**Fig. 8:**
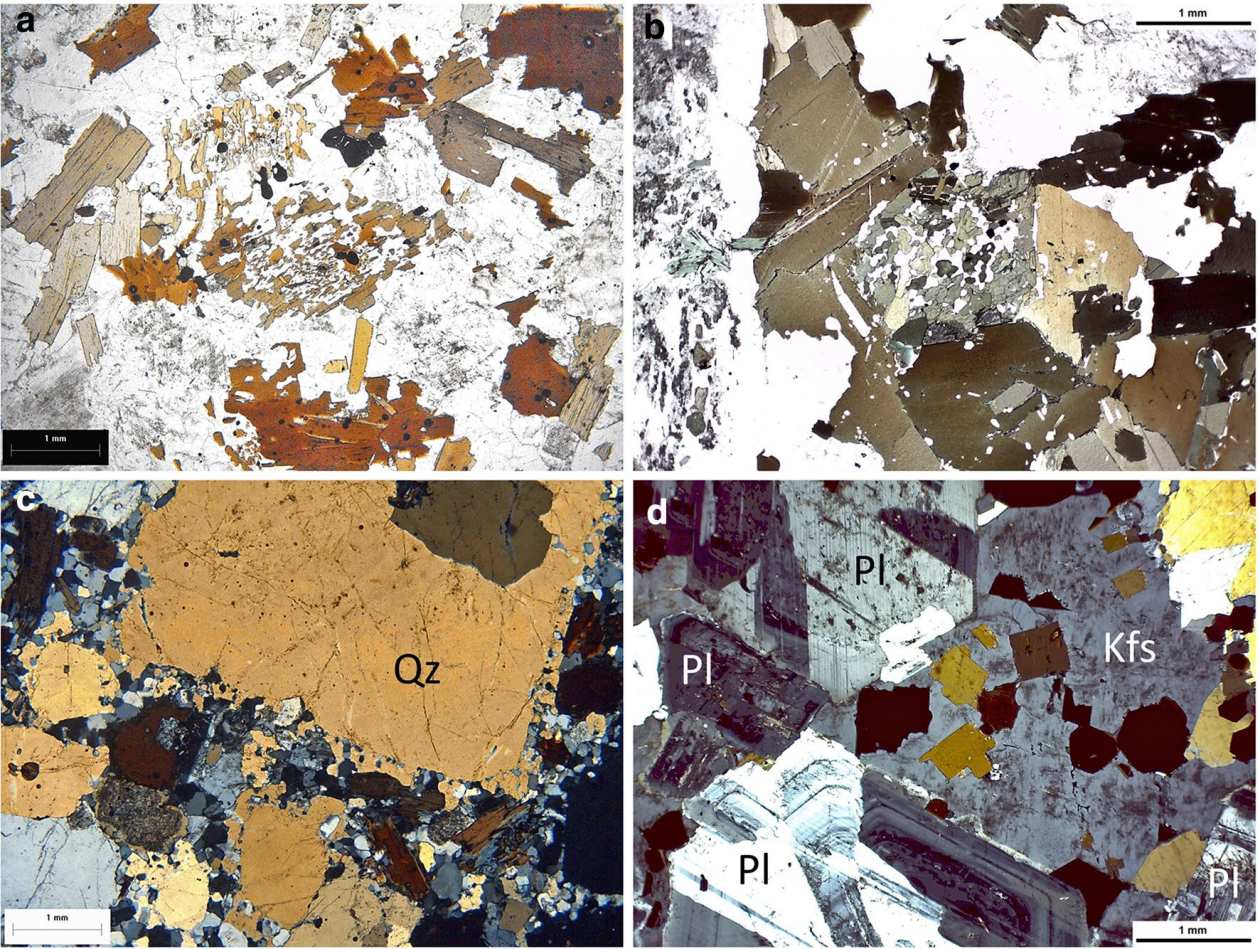
Photomicrographs illustrating the correspondence between phase-equilibrium predictions and rock textures. **a** Group of biotite–quartz pseudomorphs after orthopyroxene in S-type monzogranite sample 06MD1 from the Mount Disappointment pluton in southeastern Australia (plane light, colour version of Fig. 5a of Clemens and Benn 2010). Note the delicate texture of the fine-grained, near-solidus quartz–biotite aggregates in the centres of the pseudomorphs and the rings of coarser red–brown biotite crystals that represent earlier, higher temperature reaction with the magma. **b** Quartz–biotite pseudomorph after orthopyroxene in a granodiorite from the I-type Budduso pluton in Sardinia, Italy. The texture is similar to the pseudomorphs in **a**, but a little coarser-grained. The biotite crystals are olive-coloured and the pseudomorph lies within a magmatic segregation of larger biotite crystals. **c** Euhedral quartz phenocryst (Qz) in porphyritic micromonzogranite sample 06MD8 from the Mount Disappointment pluton (crossed polars). Note the lack of recrystallisation of the quartz and the narrow marginal zone with near-solidus outgrowths of tiny quartz grains. **d** Late-crystallised, anhedral and interstitial K-feldspar crystal (Kfs) in I-type monzogranite sample HAR1 from the Harcourt batholith in southeastern Australia. This texture is in stark contrast with the euhedral phenocrysts of K-feldspar shown in Figs. 4 and 7. Note also that the main textural skeleton of the rock is formed by plagioclase (Pl) subhedra. Crossed polars

In most crystallising granitic magmas (apart from some highly silicic alkali-feldspar granites), phase-equilibrium experiments and calculations predict that the earliest tectosilicate phase to appear on cooling is either plagioclase or quartz (in some S-type magmas). When we examine granitic rocks, we commonly find that subhedral-to-euhedral, tabular plagioclase crystals form the basic skeleton of the rock, with many of the other minerals forming interstitially to the 3D network of these plagioclase crystals (e.g., Figs. 3a, 8d). The arguments surrounding the dihedral angles between the interstitial grains and the early plagioclase crystals have already been presented. These are all features related to crystallisation from melts.

### Pyroxenes in granitic rocks

Another consistent prediction of both experimentally determined and calculated phase relations is that all but the most silicic and potassic granitic magmas crystallise a pyroxene near their liquidus (e.g., Clemens et al. 1986, 2011a, b; Clemens and Wall 1981). However, the phase diagrams also show that early formed pyroxenes will not generally survive into the subsolidus. As temperature falls and *a*H_2_O increases in the residual melts, these pyroxenes are predicted to react with the melts, to produce hydrous mafic minerals such as biotite and hornblende (e.g., Haslam 1986; Clemens and Wall 1988).

When we examine granitic rocks carefully, we commonly find small clusters of biotite or amphibole, quartz, and Fe–Ti oxide. Some such clusters are apparent in hand specimen and others are only clearly visible in thin section. In many cases, finer-grained aggregates of these minerals (some symplectic in texture) are surrounded by larger crystals of biotite and/or hornblende (e.g., Fig. 8a, b). The inner, finer-grained aggregates are inferred to be pseudomorphs after the early magmatic pyroxenes (usually orthopyroxene), formed very close to the solidus; the enveloping, larger biotite, or hornblende crystals formed at somewhat higher magmatic temperatures, with higher melt proportions present. Though common, these features are sometimes overlooked. However, the preservation of such delicate, fine-grained intergrowths, in many medium-to-coarse-grained granitic rocks, constitutes further evidence against any kind of comprehensive textural equilibration. Likewise, their presence indicates that the igneous history, predicted from phase-equilibrium studies, is mirrored faithfully in the rock textures.

### Quartz: early and late

Phase-equilibrium work on S-type granitic rocks predicts that quartz should begin to crystallise early in the solidification histories of their parent magmas. Correspondingly, when we examine the textures of many S-type granitic rocks, some of the quartz crystals (at times phenocrysts) occur as large subhedra or euhedra with square cross sections and commonly with somewhat rounded corners, clearly indicating hexagonal–bipyramidal growth habit (e.g., Fig. 8c). On the other hand, model-phase relations in less silicic I-type compositions predict the appearance of quartz only later in the crystallisation sequence. Accordingly, the textures of I-type rocks commonly show only interstitial and anhedral quartz grains (e.g., Fig. 3a) albeit, again with low dihedral angles against adjoining grains, indicating magmatic precipitation. The clear implication is that magma chemistry has controlled the stage at which quartz saturation was attained and that the quartz textures reflect igneous crystallisation.

### The K-feldspar conundrum

An important issue related to potassic alkali-feldspar occurrence in S- and I-type granitic magmas is that both experiments and phase-equilibrium modelling have shown that this mineral is typically the last major silicate to begin crystallisation; furthermore, it begins crystallisation at relatively low temperatures. The only exceptions are some highly silicic and potassic granites with low Ca contents. In such leucogranitic compositions, K-feldspar saturation occurs at considerably higher temperatures above the solidus (e.g., Clemens et al. 2011a, Fig. 5a; Clemens and Birch 2012, Fig. 16). As predicted, most K-feldspar in most granitic rocks forms interstitial and anhedral grains (e.g. Fig. 8d). Nevertheless, as we have illustrated in Figs. 4 and 7, many granitic rocks contain large subhedral-to-euhedral K-feldspar phenocrysts.

**Fig. 9:**
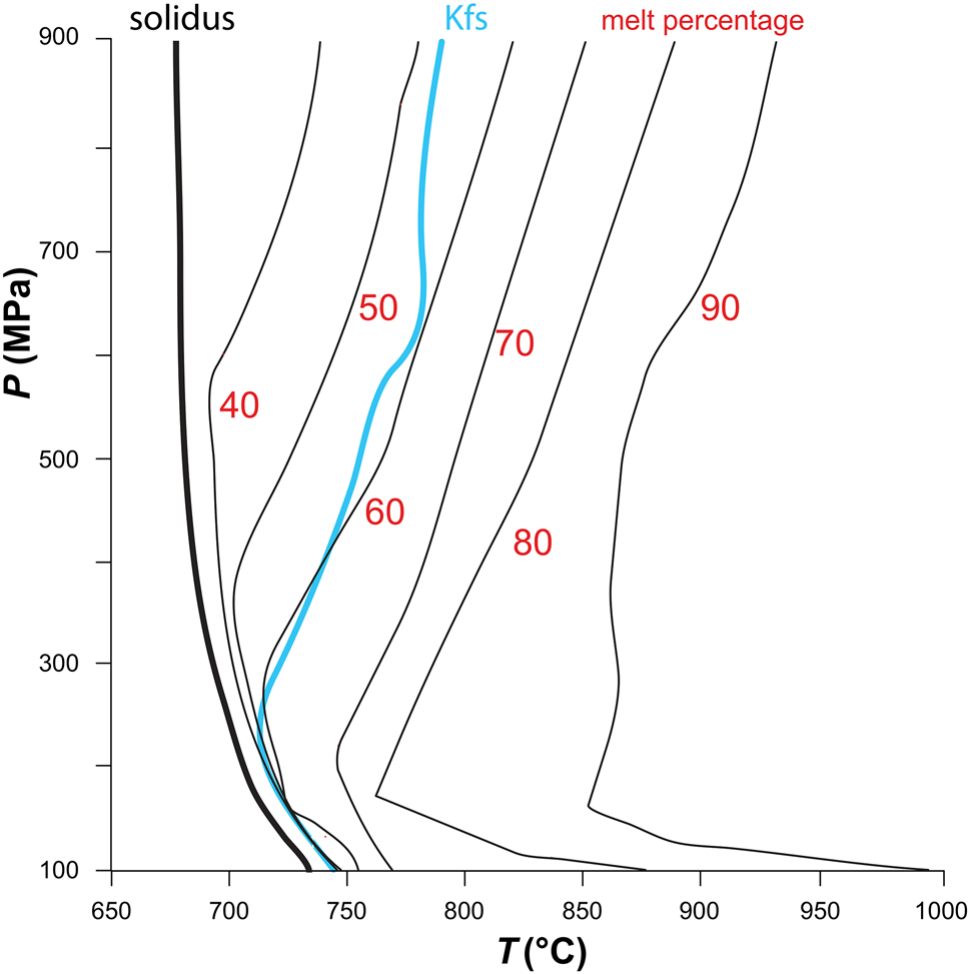
Calculated pseudosection for a magma with the silicate composition of monzogranite sample 889 (Clemens and Wall 1981), with 3 wt% H_2_O in the system. The solidus is shown as a thick black curve and the K-feldspar (Kfs) saturation boundary as a light blue curve. The diagram is contoured in melt percentage (values in red). The diagram was calculated using the software package Rcrust (Mayne et al. 2016), which uses a compiled form of meemum (a program option in PerpleX; Connolly 2009), together with the Holland and Powell (1998) thermodynamic data set, updated to version ds5.5. The bulk chemical system considered was Na_2_O–CaO–K_2_O–FeO–MgO–Al_2_O_3_–SiO_2_–H_2_O–TiO_2_ (NCKFMASHT). Activity-composition models were: biotite, garnet, and ilmenite (White et al. 2005), Opx (White et al. 2000); ternary feldspar (Cbar1 field of Holland and Powell 2003); muscovite (Coggon and Holland 2002, with their pyrophyllite and margarite end-members excluded), cordierite (Holland and Powell 1998) and haplogranitic melt (White et al. 2007). K-feldspar begins crystallisation relatively close to the solidus but with 50–60% melt present (see text for further discussion)

As an example of the relationship between the solidus and the K-feldspar saturation boundary, we present Fig. 9, which shows a calculated pseudosection for S-type monzogranite 889, studied experimentally by Clemens and Wall (1981). For clarity, the diagram has been stripped of all the mineral saturation boundaries except for that of K-feldspar, and is contoured in calculated melt percentage. In agreement with the experiments, although K-feldspar begins crystallisation relatively close to the solidus, at that stage, there is still 50–60% of melt in the system. Thus, although K-feldspar phenocrysts are not early crystallising minerals in granitic magmas, they grow in a very liquid-rich environment. This is consistent with the commonly observed short-range orientation in variable magmatic flow patterns, as shown in Fig. 7, demonstrating that such crystals can be affected by magma flow and concentrated in structures that can only have formed by such flow (e.g., Clemens 2015). Furthermore, thermal modelling of granitic pluton cooling (Bea 2010) suggests that the K-feldspar saturation boundary is achieved over a period of the order of 10^3^ years, with subsequent cooling to the solidus temperature requiring of the order of 10^4^ years. Although the growth rates of K-feldspar have only been experimentally determined for hydrous silicic melts at much greater undercoolings than may be achieved in the large plutons in which K-feldspar megacrysts occur (see, e.g., Fenn 1977; Naney and Swanson 1980), it is entirely plausible that alkali-feldspar crystals can grow to centimetric dimensions within this time frame. Thus, the commonly observed large K-feldspar phenocrysts attain their conspicuous sizes during prolonged crystallisation in abundant late-magmatic liquid.

## What did coarse-grained granitic rocks look like when first solidified?

A key difference between intrusions of widely differing volume, and hence cooling rate, is the grain size. Larger, more slowly cooled, intrusions are generally coarser-grained than their smaller equivalents. The more rapidly cooled (pressure-quenched?) roof zones of some plutons display fine-to-medium grain sizes and small phenocrysts—clearly igneous microstructures. Experimental work and inferences from natural examples (such as chill zones) suggest that this grain-size difference is a consequence of the difference in nucleation rate; higher nucleation rates in the more rapidly cooled (more highly undercooled) intrusions lead to a larger number of small crystals (e.g., Swanson 1977; Lofgren 1983; Cashman 1988; Wilhelm and Wörner 1996; Hammer et al. 1999). The corollary of this is that slower cooling rates lead to smaller degrees of undercooling, lower nucleation rates, and, therefore, fewer but larger crystals.

However, it has recently been suggested that coarse-grained plutonic rocks initially crystallised with fine grain sizes, and were subsequently coarsened by grain growth (e.g., Marsh 1998, 2007). An increase in grain size is hypothesised to occur by coarsening of small isolated grains and fine-grained aggregates, driven by the minimisation of interfacial energy, as detailed earlier. The process is suggested to involve either high-temperature grain growth or the consumption (annexation or coalescence) of some grains by others with which they come in contact during crystallisation (e.g., Mock and Jerram 2005; Marsh 2007; Hersum and Marsh 2007). Hersum and Marsh (2007) suggested that the average grain size could continue to increase through the process of grain annexation, even in cases where a little melt remains. Evidence to support this model is sparse. Marsh (1998) presented crystal-size-distribution data from the Makaopuhi lava lake that suggests loss of small grains and consequent growth of the larger grains. Hersum and Marsh (2007, their Fig. 3) presented a thin-section image of a small aggregate of olivine that purports to demonstrate the process in action. However, these images actually only show that a range of grain sizes is present in the partially solidified material.

The main argument against grain annexation and coalescence as the primary processes that result in the formation of coarse-grained textures in plutonic rocks is based on the likely extent of undercooling in large intrusions. Formation of a fine-grained starting material, with abundant crystallites, requires a significant departure from equilibrium. Large degrees of undercooling are readily generated in small igneous bodies such as lava lakes, thin sills, and dykes. However, in a slowly cooled, large pluton, the generation of a high degree of undercooling would necessitate inhibition of nucleation for a considerable time. It is unclear why or how such inhibition should occur, particularly during progressive slow solidification from the margins to the centre of a large magma sheet, in which there would be an abundance of sites for heterogeneous nucleation.

Furthermore, following the postulated abundant nucleation event, coarsening of the initially fine-grained aggregate would require slow cooling at near-equilibrium conditions, with rates specific to each mineral concerned. For example, experiments (Cabane et al. 2005) suggest that olivine coarsens more readily than plagioclase in mafic magmas. This scenario, requiring a continuous, repeated, and spatially localised oscillation between high undercooling and low undercooling, is unlikely to occur in deep-seated plutons. Note, however, that a contrasting situation occurs in pegmatites, in which strong undercooling, consequent delayed nucleation and eventual rapid growth of very large crystals, are promoted by fluxes of volatile components, despite very rapid cooling of these small intrusions (London 2008; Nabelek et al. 2010).

## Conclusions

The evolution of microstructures in solidifying (crystallising) systems is well understood. Numerous observations of microstructures that develop during materials manufacture, experimental simulations of solidification of silicate melts, and comparisons with natural examples provide a clear picture of the contrast between microstructures that form during crystal growth and reaction and those controlled by the minimisation of interfacial energies. The underlying physics governing this evolution is also well understood and, although we currently have comparatively little information on the rates at which these changes occur, we can make comparisons with microstructures in metamorphic rocks, to place some temporal constraints on the processes.

We suggest that the general absence of a significant microstructural modification in granitic rocks results from three factors. The first is the lower temperatures and narrower temperature ranges over which cooling occurs in granitic magmas, in contrast to the situation in higher temperature mafic magmas, many of which do show the evidence of subsolidus grain-shape adjustment (Vernon 2004, 2010; Holness and Vernon 2014). The second is that, as a consequence of the chemical compatibility of the granitic rock mineral assemblage, there is no chemical driving force for recrystallisation (Vernon and Paterson 2008, p. 47). Finally, the general absence of significant solid-state deformation of granitic mushes means that there is no driving force for recrystallisation caused by the high internal energies that are associated with lattice distortions (Vernon and Paterson 2008, p. 47).

Our detailed examination of a wide range of microstructures preserved in granitic rocks shows no widespread evidence for either the kinds or degrees of subsolidus textural modifications advocated by Coleman et al. (2005), McBirney (2009), Glazner and Boudreau (2011), Glazner et al. (2017), and Bartley et al. (2018). Instead, microstructures are overwhelmingly those created during igneous crystallisation, and, therefore, provide an excellent record of phase equilibria and cooling rates. In general, therefore, microstructures in granitic rocks do not deceive. As a caveat, some plutons have undergone fluid-driven alteration, some have undergone shear deformation, and others have been affected by post-solidification metamorphism. Thus, each granitic pluton should be examined with reference to its specific geological setting and history.
